# On the Aggregation of Apolipoprotein A-I

**DOI:** 10.3390/ijms23158780

**Published:** 2022-08-07

**Authors:** Rebecca Frankel, Emma Sparr, Sara Linse

**Affiliations:** 1 Biochemistry and Structural Biology, Lund University, P.O. Box 124, SE22100 Lund, Sweden; 2 Division of Physical Chemistry, Lund University, P.O. Box 124, SE22100 Lund, Sweden

**Keywords:** apolipoprotein A-I, aggregation, condensates, plaques

## Abstract

In vivo, apolipoprotein A-I (ApoA-I) is commonly found together with lipids in so-called lipoprotein particles. The protein has also been associated with several diseases—such as atherosclerosis and amyloidosis—where insoluble aggregates containing ApoA-I are deposited in various organs or arteries. The deposited ApoA-I has been found in the form of amyloid fibrils, suggesting that amyloid formation may be involved in the development of these diseases. In the present study we investigated ApoA-I aggregation into amyloid fibrils and other aggregate morphologies. We studied the aggregation of wildtype ApoA-I as well as a disease-associated mutant, ApoA-I K107Δ, under different solution conditions. The aggregation was followed using thioflavin T fluorescence intensity. For selected samples the aggregates formed were characterized in terms of size, secondary structure content, and morphology using circular dichroism spectroscopy, dynamic light scattering, atomic force microscopy and *cryo* transmission electron microscopy. We find that ApoA-I may form globular protein-only condensates, in which the α-helical conformation of the protein is retained. The protein in its unmodified form appears resistant to amyloid formation; however, the conversion into amyloid fibrils rich in β-sheet is facilitated by oxidation or mutation. In particular, the K107Δ mutant shows higher amyloid formation propensity, and the end state appears to be a co-existence of β-sheet rich amyloid fibrils and α-helix-rich condensates.

## 1. Introduction 

Atherosclerosis (also called arteriosclerotic vascular disease) is linked to deposits—plaques—in arteries, causing lesions and narrowing of these vessels. The plaques contain apolipoprotein A-I (ApoA-I), lipids, macrophage cells and other components as reviewed [1,2]. The plaques build up over many years and although the exact cause is unknown, co-aggregation of ApoA-I and lipids seems to be involved [3]. Moreover, deposited ApoA-I has been found in the form of amyloid fibrils [4], suggesting that amyloid formation may be involved in analogy with a class of human diseases [5,6,7,8].

The healthy function of ApoA-I involves the formation of high-density lipoprotein (HDL) and other lipoprotein particles that transport lipids in blood and cerebrospinal fluid (CSF) [9]. HDL particles typically have a discoidal shape with a diameter of 7–13 nm [10] and are built up by a patch of a lipid bilayer with the lipid acyl chains at the edges shielded from water by a circumference belt of α-helical ApoA-I [11,12]. While the exact molecular triggers of the conversion from healthy lipid-protein co-aggregates, HDL particles, to pathological aggregates, plaques, is not yet understood, lipid oxidation and the concentration of lipids, including cholesterol, seems to play a role [13,14,15,16]. Other identified factors are mutations and truncations of the ApoA-I sequence. One example of changes observed in atherosclerosis patients is ApoA-I_Helsinki_, with the deletion of residue 107 (K107Δ) [17]. The K107Δ variant has been detected in amyloid lesions, however, in different organs compared to another disease-associated variant G26R, suggesting that different structural features or different susceptibilities to micro environmental factors could determine the pathogenicity of these and other ApoA-I mutants [17].

Studies of ApoA-I aggregation into the larger objects that are possibly associated with plaque formation have largely been focused on the amyloid fibril formation. Still, there remain some apparent inconsistencies in the literature. Some studies report on the fibril formation propensity of wildtype, full length ApoA-I [18], while others found such property only for ApoA-I variants caused by proteolytic truncation [19], oxidation of the methionine residues [20,21,22], sequence substitutions [17,23,24,25], or the two latter combined [26]. 

**Figure 1:**
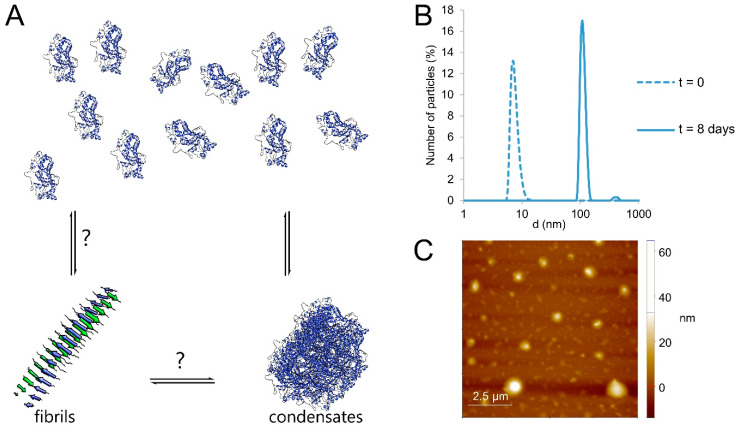
(**A**) Possible forms of aggregated ApoA-I, with α-helical ApoA-I in globular condensates or β-sheet rich amyloid fibril structure, both of which may be in equilibrium with the monomer. (**B**) Number-based size distribution derived using dynamic light scattering (DLS) for an ApoA-I sample directly after isolation by size-exclusion chromatography (SEC; t = 0) and after 8 days incubation at 37 °C. (**C**) AFM image of the same sample after 8 days.

In the present study, we have investigated ApoA-I aggregation in a broad sense, considering amyloid aggregates as well as other aggregate morphologies (Figure 1). We studied the aggregation behavior of wildtype ApoA-I as well as a disease-associated mutant, ApoA-I K107Δ, in different solution conditions. For the conditions under which aggregation was observed, the formed aggregates were characterized with respect to size, morphology, and protein conformation using a combination of optical spectroscopy, dynamic light scattering and cryo-electron microscopy imaging. This combination of methods allows the distinction between two major types of morphologies that are globular condensates where the protein retains the α-helical conformation, and β-sheet rich amyloid fibrils (Figure 1). 

## 2. Results

We have investigated ApoA-I self-assembly, and in particular, under what conditions this protein forms larger aggregates. The aggregates formed may have different morphologies, including amyloid fibrils and globular condensates (Figure 1). The aggregation may or may not involve changes in protein secondary structure. In order to gain insights into the conditions under which ApoA-I forms amyloid fibril, we varied the system in terms of solution conditions, protein oxidation and protein mutation (Table S1). To differentiate between the different types of aggregate structure, we utilized a range of complementary biophysical techniques. From the dynamic light scattering (DLS) measurements, we can detect the presence of larger aggregates and obtain an estimate of their diameter. To distinguish between the different aggregate morphologies, we used cryo-EM and AFM. Motivated by previous studies showing that free ApoA-I as well as ApoA-I in HDL particles exhibits α-helical structure [4,27,28], while the protein in amyloid fibrils exhibits cross-β structure [29], we here investigated the samples containing protein aggregates by means of circular dichroism (CD) spectroscopy. Finally, we studied the thioflavin-T (ThT) fluorescence intensity over time. The ThT probe is commonly used as an indirect tool to detect amyloid formation [30,31], and has previously been used in studies of ApoA-I amyloid aggregation [4,20,22,25]. 

### 2.1. Self-Assembly of ApoA-I and the Effects of Changing Extrinsic and Intrinsic Factors

First, we investigated the aggregation behavior of wildtype ApoA-I in buffer solution at pH 7.4. For all experiments presented, we started from a sample composed of freshly purified ApoA-I. While the scattered intensity in DLS is strongly dependent on particle size [32], the method can be used to monitor charges in self-assembly state in a solution. This initial state samples were characterized using DLS, giving a diameter of ca. 6 nm (Figure 1B), which is approximately the size expected for the monomeric protein. The same samples were characterized again after incubation for 6–8 days at 25 °C in 20 mM sodium phosphate, 0.2 mM EDTA, pH 7.4. Unless otherwise specified, the samples were incubated without agitation. The incubation time was chosen based on DLS experiments showing drastic changes in aggregate size after 4–5 days (Figure S1), while at later timepoints there are no significant further changes. After 8 days, DLS reveals the emergence of objects of slightly more than 100 nm diameter assuming spherical aggregate shape, with a broad size distribution (Figure 1B). These estimates were further supported by the AFM images of dried aggregates deposited on flat mica surfaces (Figure 1C), showing close to globular particles with diameters between 100 and 1000 nm. Finally, no increase of ThT fluorescence could be observed for the duration of 6 days (Figure S2), which would be expected for ApoA-I amyloid fibrils according to previous reports [4,20,22,25]. 

**Figure 2:**
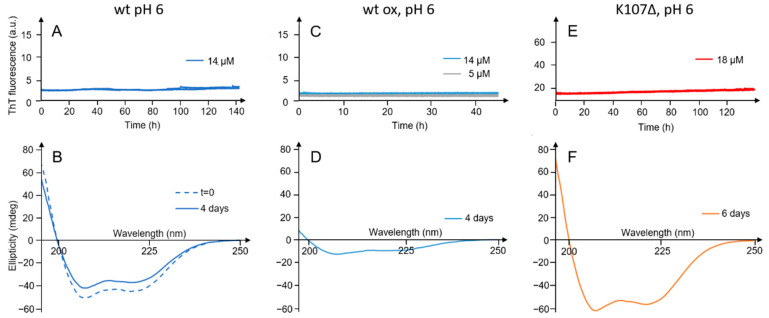
Time evolution of ThT fluorescence and CD spectra at pH 6.0. ThT fluorescence intensity versus time measured in duplicates in the presence of 14 µM ApoA-I wt (**A**), 5 and 14 µM oxidized ApoA-I wt (**C**) and 18 µM K107Δ ApoA-I (**E**). CD spectra recorded for 11 µM wt (**B**), 5 µM oxidized wt (**D**) and 18 µM K107Δ (**F**) at time zero (0 h) or at a later stage (4 or 6 days) as indicated in each panel.

**Figure 3:**
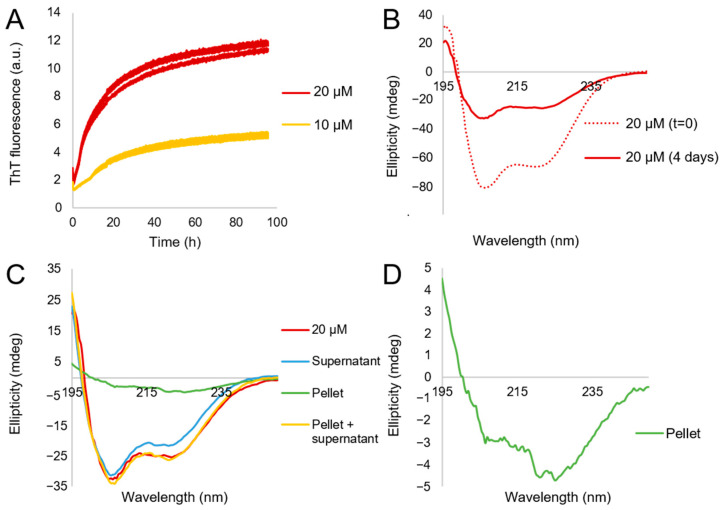
Time evolution of ThT fluorescence and CD spectra of the ApoA-I mutant K107Δ in phosphate buffer at pH 6.0. (**A**) The ThT fluorescence intensity over time for 10 and 20 µM protein in triplicates. The initial decrease in ThT fluorescence signal is due to temperature stabilization. (**B**) The observed CD spectrum for the initial state (t = 0, dotted line) and at a later stage (4 days, red line). (**C**) The observed CD spectra after separation of the soluble (blue) and insoluble fractions (green). The 4 days sample from panel (**B**) (red) is included for comparison. The two spectra of the supernatant and the pellet were summed (yellow), to compare their superposition to the spectrum of the non-separated sample. (**D**) Zoom-in on the pellet sample spectrum in (**C**), to observe this weak spectrum more easily.

Next, we started a broad scouting to investigate the aggregation behavior of ApoA-I varying both extrinsic or intrinsic conditions. The conditions investigated were based on reports on ApoA-I amyloid formation [4,17,20]. We investigated the effects of decreasing pH, methionine residue oxidation, and mutations (K107Δ). We further studied the combination of the different factors, by either lowering the pH, as well as oxidizing the methionine residues, or using a more aggregation prone mutant (or K107Δ), or all of them together (Figure 2 and Figures S3–S5). We also investigated the effect of combining oxidation of the methionine residues with shaking during the incubation time (Figure S6). As a tool to screen for the conditions under which ApoA-I may form amyloid fibrils, we measured the ThT fluorescence, and all the conditions studied are summarized in Table S1. Out of all samples investigated, only one shows a clear increase in the ThT signal, thus indicating amyloid formation. This sample was the mutant K107Δ (6–20 µM in 20 mM sodium phosphate, 0.2 mM EDTA) at reduced pH and under oxidizing conditions (Figure S5), which is characterized below in more detail (Figure 3). In addition, a slight increase in ThT fluorescence was found for the wildtype ApoA-I (18 µM in 20 mM sodium phosphate, 0.2 mM EDTA) at reduced pH under shaking conditions (Figure S6).

### 2.2. Self-Assembly of the ApoA-I Mutant K107Δ

The samples containing aggregates formed by the ApoA-I mutant K107Δ after oxidation, and at pH 6.0 were studied by means of CD spectroscopy. Although this method is associated with perturbing factors and large uncertainties for the quantification of the secondary structure content in a sample [33,34], it is highly useful for monitoring changes in secondary structure within or between samples. A first inspection of the CD spectra in Figure 3B indicates α-helical secondary structure in both the initial and aggregated states. In addition, the overall signal intensity is lower for the aggregated samples as compared to the initial sample, suggesting sedimentation of larger objects. The sedimentation was also observed through ocular inspection. However, as the ThT fluorescence increased over time (Figure 3A), and as this has previously been reported for ApoA-I of β-sheet structure [20], we investigated whether the precipitating aggregates give the same or a different CD spectrum compared the protein in solution, or if there is a co-existence of different conformations in the sample. 

Because the ThT fluorescence intensity increased over time (Figure 3A), we suspected that the aggregates might contain β-sheet structure even though this was not apparent in the raw CD spectra. We therefore investigated the specific contribution to the CD signal from the fraction consisting of precipitating aggregates by separating the supernatant from the aggregates by centrifugation. The supernatant was transferred to a new vessel, while the pellet was dispersed in buffer, and CD spectra were recorded for both samples. This analysis clearly shows that, while the intensity of the CD signal at 208 nm of the supernatant is close to that from the full non-separated sample, the spectral shapes differ. While the fraction in the supernatant seems to contain mainly α-helical structure as inferred from the double minima in the CD spectrum (Figure 3C), it is clear that the minimum at 222 nm is deeper for the non-separated sample compared to the supernatant. The dispersed pellet, however, gives rise to a spectrum with a single minimum close to 222 nm—more clearly observable when expanding the y-axis (Figure 3D), indicative of β-sheet structure. There is a shoulder at 206 nm in Figure 3D), which likely originates from traces of supernatant left in the sample. For the linear combination of the spectra from the separated samples, both the intensity and the overall shape of the non-separated sample is replicated (Figure 3C). 

**Figure 4:**
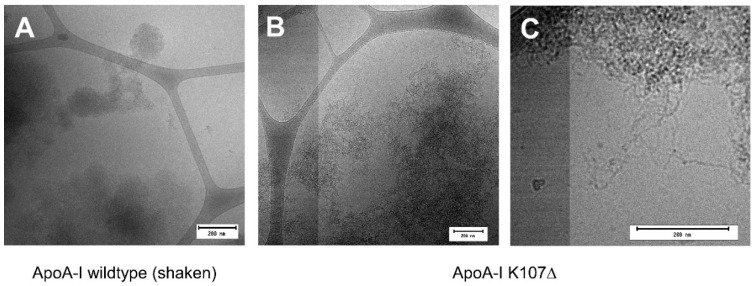
Cryo-TEM images of oxidized (**A**) wildtype ApoA-I, aggregated under agitation, and (**B,C**) K107Δ ApoA-I. Both samples were aggregated in buffer with pH 6.0. For (**B,C**), the co-existence of larger, more compact aggregates (**B**), as well as more fibrillar structures (**C**) and single fibrils (Figure S7C,D) could be observed. The scale bar in each image is 200 nm (note the difference in magnification of the images).

Finally, the morphology of the aggregates formed by the oxidized K107Δ mutant at pH 6.0 was investigated using cryo-TEM (Figure 4), because this method has been shown to be superior to negative staining TEM in maintaining the amyloid structures in the sample [35]. As oxidized wildtype ApoA-I at pH 6.0 and after agitation showed a slight increase in ThT signal (Figure S6), this sample too was imaged with cryo-EM. In all samples, we observe large, compact aggregates of different sizes. Fibrillar structures are observed in the sample containing the K107Δ mutant, both as part of the compact aggregates (Figure 4C and Figure S7A,B) and as isolated fibrils (Figure S7C,D).

## 3. Discussion

The results of a range of complementary methods imply that ApoA-I readily aggregates into larger microscopic structures. The aggregates formed by the wildtype ApoA-I in buffer at pH 7.4 are mainly globular in shape, and the α-helical protein conformation present for the monomeric state is retained also in the aggregates. This is distinct from the β-sheet rich amyloid fibrillar structures that have previously been reported for certain solution conditions, protein modifications [20] and mechanical agitation [22]. For the more aggregation prone mutant ApoA-I K107Δ, we find a co-existence of amyloid fibrils and globular condensates after oxidation and when present at mildly acidic solution conditions. 

The aggregation of proteins with retained secondary structure into large and polydisperse globular condensates signifies a system with relatively low colloidal stability. This aggregated state does not have to be the lowest free energy-state, i.e., an equilibrium structure, but may be kinetically stable. Changes in protein charge through mutation or variations in pH may alter the propensity for aggregation via the long-range electrostatic repulsive interactions. In the case when the protein aggregates into amyloid fibrils, it is not only a question of colloidal stability, as the aggregation process also involves changes of molecular scale structures. For ApoA-I, it is then necessary to consider both the destabilization of the native α-helical state of the protein in solution, and the balance of attractive and repulsive interactions in the near-crystalline packing of monomers in β-sheet conformation in the amyloid fibrils. 

In the present study, we explore a range of extrinsic and intrinsic factors of the system to identify conditions under which ApoA-I forms globular condensates versus amyloid fibrils (Table S1). Changes in protein charge and protein hydrophobicity may alter the colloidal stability as well as the stability of native α-helical conformation relative to the β-sheet rich amyloid structure. In the latter case also the protein concentration comes into play as we are comparing unimolecular folding with multi-molecular assembly. The pI of wildtype ApoA-I is approximately 5.5, depending on its isoform [36]. A decrease in pH would thus lead to a reduced net repulsion between the proteins in solution, as well as between side chains within the protein, likely allowing for a different conformation. Through the K107Δ mutation, one lysine has been deleted, again altering the net charge of the protein. This could further lead to a disruption of α-helical structure, if the lysine is involved in stabilizing interactions. Moreover, the deletion of one residue in a helix perturbs its amphipathic pattern, which likely also affects the protein-only condensates. The stability of both folded structures, condensates and fibrils, can also be affected by protein modification that imposes steric effects on their molecular packing. Oxidation of the methionine residues creates methionine sulfoxide, where a carboxyl is added to the sulfur of methionine, which may differentially influence the stability of the condensates and fibrils due to increased steric hindrance in one or both cases. It could also make the helical conformation less favorable due to a change in hydrophobicity of the side chain. All three methionines that may be oxidized—M86, M112, and M148—are suggested to be located in different helices in wildtype ApoA-I, meaning that the oxidation of any one may influence the stability of the protein [23,37]. Oxidation could further affect the system due to a shift in polarity and possibly the pK_a_ of surrounding amino acid residues. 

Out of all the systems investigated here, there is only one case where we observe clear amyloid formation, and that is for the K107Δ mutation after oxidation and at mildly acidic pH. As discussed above, all these modifications likely lead to destabilization of the native α-helical conformation of ApoA-I. As none of the above on their own was shown to lead to amyloid fibril formation, most likely the combination of all of them lead to a cumulative effect large enough to convert the ApoA-I conformation towards the amyloid β-sheet structure. Still, within the time-frame of the experiment, the amyloid fibrils are not the prevailing aggregate structure even after all these modifications, as there is a co-existence between amyloid fibrils and globular condensates with retained α-helical structure. As both these aggregate structures cannot represent the equilibrium state, it is further possible that conformational conversion will occur within already formed aggregates if the energy barrier is low enough [38], although this cannot be captured in our experiments. 

The characterization of the ApoA-I aggregation behavior in different conditions may deepen the understanding of ApoA-I assembly and aggregation in vivo. ApoA-I is detected both in arteriosclerotic plaques and in amyloidal deposits in different organs. In vivo, ApoA-I may undergo oxidation in the oxidative environments of plaques or from myeloperoxidase, an enzyme involved in atherosclerosis [39,40], leading to amyloid fibril formation [21,22]. Mutations and oxidation can also affect the susceptibility to proteases. Thereby it is conceivable that the aggregation of ApoA-I is carried out differently, resulting in different aggregate structures, depending on the disease and part of the body. 

## 4. Materials and Methods

Chemicals. All chemicals used for buffers were of analytical grade purchased from Sigma Aldrich. 

### 4.1. Expression and Purification of Recombinant Human ApoA-I

The expression and purification of ApoA-I was carried out as reported [41]. In summary, human ApoA-I was expressed in *E. coli* in fusion with the autoprotease N^pro^ mutant EDDIE, on which a His-tag was added. The formed inclusion bodies were extracted from the cells by sonication, collected and washed through 5 centrifugation and sonication steps. The inclusion bodies were dissolved in 4 M urea and the His-EDDIE refolded and cleaved from the target protein by dilution into 5 mM dithiothreitol (DTT), 10 mM Tris/HCl, 1 mM ethylenediaminetetraacetic acid (EDTA), pH 8.0 and left on stirring for 48 h. The cleaved ApoA-I was purified through anionic ion exchange, immobilized metal affinity chromatography, hydrophobic interaction chromatography and boiling prior to being aliquoted, lyophilized and stored at −20 °C. Because the sample elutes in water at the last step, the lyophilized protein is salt-free except for the counter-ions [41]. The same protocol was used for wildtype ApoA-I, and a natural single point mutant, ApoA-I K107Δ [41].

### 4.2. Preparation of ApoA-I Samples

Aliquots of purified ApoA-I were dissolved in 6 M GuHCl, and the monomer was isolated by size exclusion chromatography (SEC) on a 10 mm × 300 mm ENrich™ SEC70 column (Bio-Rad) in 20 mM sodium phosphate, 0.2 mM EDTA, pH 7.4 or 6.0. After the SEC step, no other proteins were observed by SDS PAGE [41]. The protein concentration was determined from the absorbance of either the integrated peak area or of the collected fraction using a NanoDrop 2000 instrument (Thermo Scientific) and the concentration determined from the baseline-corrected absorbance at 280 nm using ε_280_ = 1.13 L g^−1^ cm^−1^ [42] and molecular weight 28 kDa. 

### 4.3. Oxidation Using H_2_O_2_

Oxidation of the methionine residues of ApoA-I was performed as described elsewhere [22]. In summary, H_2_O_2_ from a 30% (9.8 M, according to Sigma Aldrich, St. Louis, MO, USA) stock was added in a 1000:1 H_2_O_2_:protein molar ratio and the samples incubated at 37 °C overnight. The solutions were transferred to individual Slide-A-Lyzer™ MINI Dialysis Devices (MWCO 3.5 kDa; Thermo Scientific, Waltham, MA, USA) and dialyzed against 20 mM sodium phosphate, 0.2 mM EDTA, pH 6.0, with stirring at 4 °C for 24 h. The buffer was changed thrice.

### 4.4. Shaking

For agitation, samples were either transferred to Eppendorf tubes and incubated at 37 °C with orbital shaking at 800 rpm, or subjected to orbital shaking between reads in a plate reader. The control samples were treated in the same way but incubated without agitation. 

### 4.5. Thioflavin-T Fluorescence Intensity

The monomer generated as above was supplemented with 10 μM thioflavin T (ThT, Calbiochem) from a 2 mM stock (in water, filtrated through 0.2 µm filter). Each sample was pipetted into wells of a 96 well half-area plate of black polystyrene with a clear bottom and PEG coating (Corning 3881, Glendale, AZ, USA) in triplicates, 100 μL per well. Aggregation was initiated by placing the 96-well plate at 37 °C under quiescent conditions in a plate reader (Fluostar Omega or Fluostar Optima BMGLabtech, Offenburg, Germany). The ThT fluorescence [31] was measured through the bottom of the plate every 60 s with a 440 nm excitation filter and a 480 nm emission filter. 

### 4.6. Circular Dichroism Spectroscopy

Circular dichroism (CD) spectra [43] were recorded using a JASCO J-815 CD spectrometer with a JASCO PTC-423S/17 Peltier type thermostated cell holder. The secondary structure of the proteins was evaluated by far-UV CD spectroscopy, with spectra recorded between 195 and 250 nm, 20 nm/min, D.I.T. 8 s, bandwidth 1 nm, 3 accumulations, in a 2 mm cuvette at 20 °C. The protein concentrations were typically in the range 7–15 µM, corresponding to 200–420 µg/mL, as determined from the baseline-corrected absorbance at 280 nm using ε_280_ = 1.13 L g^−1^ cm^−1^ [42] and molecular weight 28 kDa.

### 4.7. Dynamic Light Scattering

The samples were transferred to quartz cuvettes (3 mm path length). Dynamic light scattering (DLS) measurements were performed using a Malvern Zetasizer Nano-S (Malvern Panalytical, Malvern, UK), software version 7.13, with set angle 173°and temperature 20 °C. Data analysis was done using the Malvern Zetasizer software to obtain the apparent intensity-based and number-based size distributions. Using the Stokes–Einstein equation [44,45] assuming spherical particles, the apparent hydrodynamic radius was calculated. The number-based size distributions calculated by the software assuming, from Raleigh’s approximation [32], that the intensity of scattering by a particle is proportional to the sixth power of its radius. 

### 4.8. Cryo Transmission Electron Microscopy (Cryo-TEM)

Aliquots of ApoA-I wildtype, and K107Δ were oxidized with H_2_O_2_ as described above. The wildtype sample was agitated in a low-binding tube (Axygen) at 800 rpm for 120 h at 37 °C to investigate the effect of shaking, while the K107Δ sample was incubated in wells of a PEGylated 96-well plate (corning 3881) in a plate reader for 120 h at 37 °C validated using ThT fluorescence to monitor fibril formation. Specimens for electron microscopy [35] were prepared in a controlled environment vitrification system (CEVS) as thin liquid films on lacey carbon filmed copper grids and plunged into liquid ethane at −180 °C, and stored under liquid nitrogen. The specimens were transferred using a Fischione Model 2550 cryo transfer tomography holder into the electron microscope, JEM 2200FS (JEOL, Tokyo, Japan), equipped with an in-column energy filter (Omega filter; this filter is composed of four electromagnets and has the shape of the letter Ω) between the intermediate and projector lenses of the microscope [46]. The acceleration voltage was 200 kV and zero-loss images were recorded digitally with a TVIPS F416 camera (TVIPS, Gauting, Germany) using SerialEM under low dose conditions with a 30 eV energy selecting slit in place.

### 4.9. Atomic Force Microscopy

For atomic force microscopy (AFM) [47], 10 µL of samples were pipetted onto mica plates and dried overnight. They were imaged on an AFM—Park XE 100 (Park Systems, Suwon, Korea) in intermittent (non-contact) mode using a PointProbe^®^ Plus probe (Park Systems) with a frequency of ~330 kHz, and a force constant of 42 N/m.

## 5. Conclusions

The results of the current work show that ApoA-I may form globular protein-only condensates, in which the α-helical conformation of the protein is retained. The conversion to amyloid fibrils rich in β-sheet is facilitated by oxidation or mutation. In particular, the K107Δ mutant shows higher amyloid formation propensity, and the end state appears to be a co-existence of β-sheet-rich amyloid fibrils and α-helix-rich condensates. 

## Data Availability

All data presented will be made available upon reasonable request to the corresponding author.

## Supplementary Materials

The following material can be downloaded at https://www.mdpi.com/article/10.3390/ijms23158780/s1.
