# Supplementary material for: On the Aggregation of Apolipoprotein A-I
Source: Int J Mol Sci. 2022 Aug 7;23(15):8780. doi: 10.3390/ijms23158780 (PMC9369196; doi:10.3390/ijms23158780)
Supplement: Supplementary file 1 [file ijms-23-08780-s001.zip › ijms-1825447-supplementary.pdf]

On the aggregation of apolipoprotein A-I

Rebecca Frankel[1,2], Emma Sparr[2], Sara Linse[1]

1) Biochemistry and Structural Biology, Lund University, P O Box 124, SE22100 Lund, Sweden
2) Division of Physical Chemistry, Lund University, P O Box 124, SE22100 Lund, Sweden

Supporting Information

Table S1. **Experimental conditions.** Summary of the extrinsic or intrinsic conditions under which ApoA-I amyloid formation was investigated. Relative to wildtype ApoA-I at pH 7.4, the altered parameters were decreased pH, oxidation of methionine residues, point mutation (K107Δ), and agitation of the samples. The samples were monitored with ThT fluorescence, and some samples were further characterized using DLS (with diameter calculated assuming spherical particles), CD, or imaging with AFM or cryo-EM.

Condition	Concentration	Vessel	# of replicates	Agitation	ThT fluorescence increase	Estimated aggregate diameter (DLS)	Observed morphology	Dominant CD spectrum
pH 7.4*	5, 10, 20 µM<br>6 µM	Plate then E-tube<br>NB-tube	3<br>1	-	No increase	100-1000 nm	Globules†	α-helical
pH 6.0	14 µM	Plate	2	-	No increase	N/A	N/A	α-helical
Oxidation, pH 7.4	10 µM	Plate	3	-	No increase	N/A	N/A	N/A
Mutation K107Δ, pH 7.4	7.8, 12, 24 µM	Plate	3	-	No increase	N/A	N/A	N/A
Oxidation, pH 6.0	5, 14 µM<br>10 µM<br>10.7 µM	Plate<br>E-tube<br>E-tube	3<br>1<br>1	-	No increase	100-1000 nm	Globules†	α-helical
Oxidation, shaking, pH 6.0	10 µM<br>7.1 µM<br>17.5 µM<br>10.7 µM<br>7.1 µM	Plate/E-tube<br>E-tube<br>E-tube<br>E-tube<br>E-tube	2/1<br>1<br>1<br>1<br>2	Shaking<br>Shaking<br>Shaking<br>Stirring<br>Shaking	Slight increase	500-1000 nm	Globules‡	α-helical
Mutation K107Δ, pH 6.0	17 µM	Plate/E-tube	2/1	-	No increase	N/A	N/A	α-helical
Mutation K107Δ, oxidation, pH 6.0	6.4, 12.8 µM<br>7.3 µM<br>10, 20 µM	Plate<br>Plate<br>Plate	2<br>2<br>3	-	Increase	N/A	Co-existence of fibrils and globules	α-helical/β-sheet

*while all other experiments were performed are at 37 ˚C, the experiments at this condition were repeated at 25 ˚C

† imaged by AFM

‡ imaged using both AFM and cryo-EM

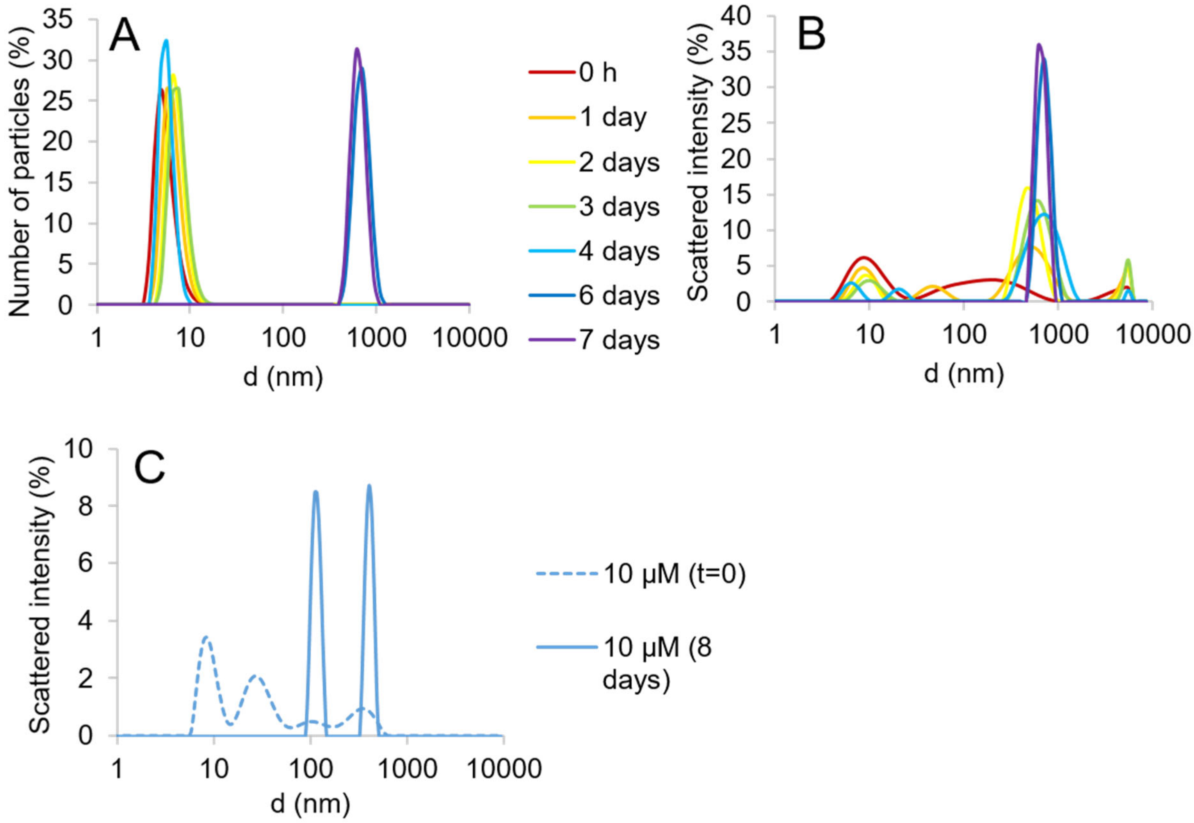

Figure S1. DLS data for wt ApoA-I at pH 7.4. A) The number-based size distribution calculated from the data acquired once per day (except for day 5) for up to 7 days at 25 °C. A clear increase in size can be observed after 4-6 days. B) The intensity-based distributions calculated from the same data as in A). C) The intensity-based size distributions calculated for the DLS data shown in Figure 1.

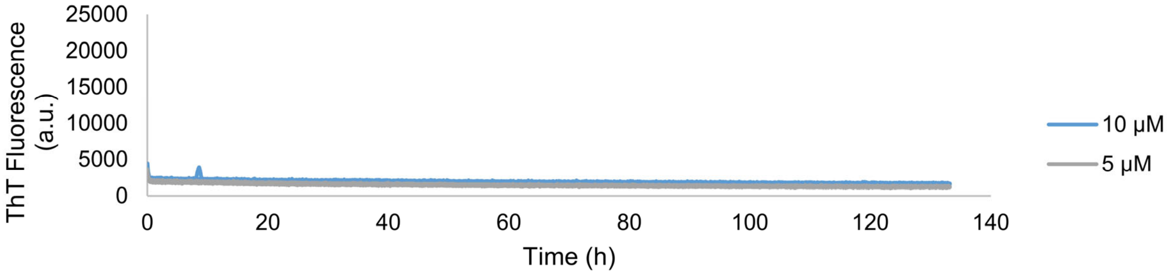

Figure S2. The aggregation of wt ApoA-I at pH 7.4. Fluorescence intensity as a function of time for ThT in the presence of wild-type ApoA-I at pH 7.4, at 37 °C, in at two concentrations measured in triplicates.

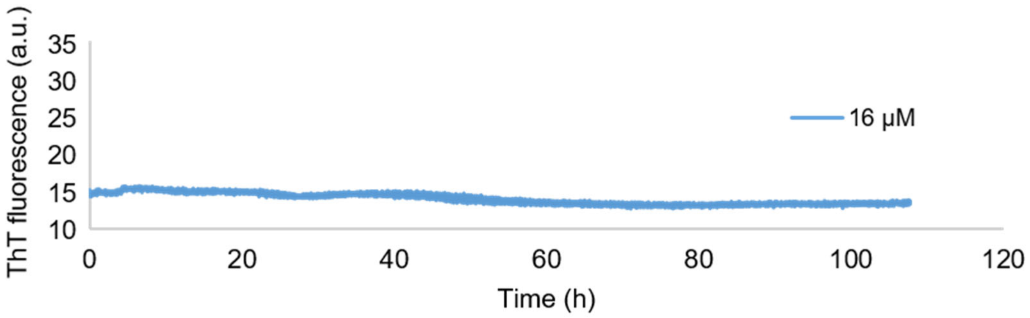

Figure S3. The aggregation of oxidized wt ApoA-I at pH 7.4. Fluorescence intensity as a function of time for ThT in the presence of 16 µM wild-type ApoA-I after methionine oxidation at pH 7.4, at 37 °C, in duplicate.

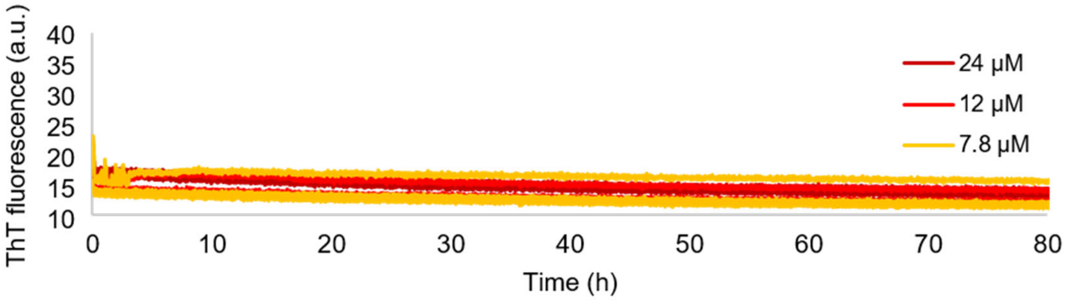

Figure S4. The aggregation of K107Δ ApoA-I at pH 7.4. Fluorescence intensity as a function of time for ThT in the presence of K107Δ ApoA-I at pH 7.4, 37 °C, at three concentrations in triplicates.

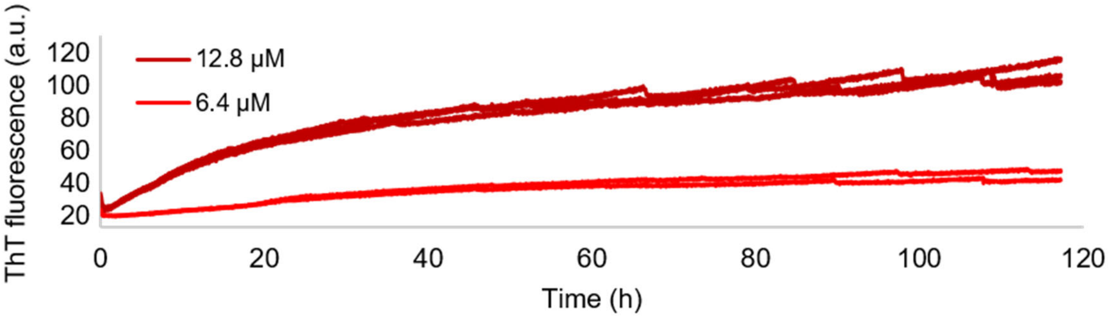

Figure S5. The aggregation of oxidized K107Δ ApoA-I at pH 6.0. ThT fluorescence intensity as a function of time for ApoA-I mutant K107Δ after methionine oxidation at pH 6.0, 37 °C, at two concentrations measured in triplicates.

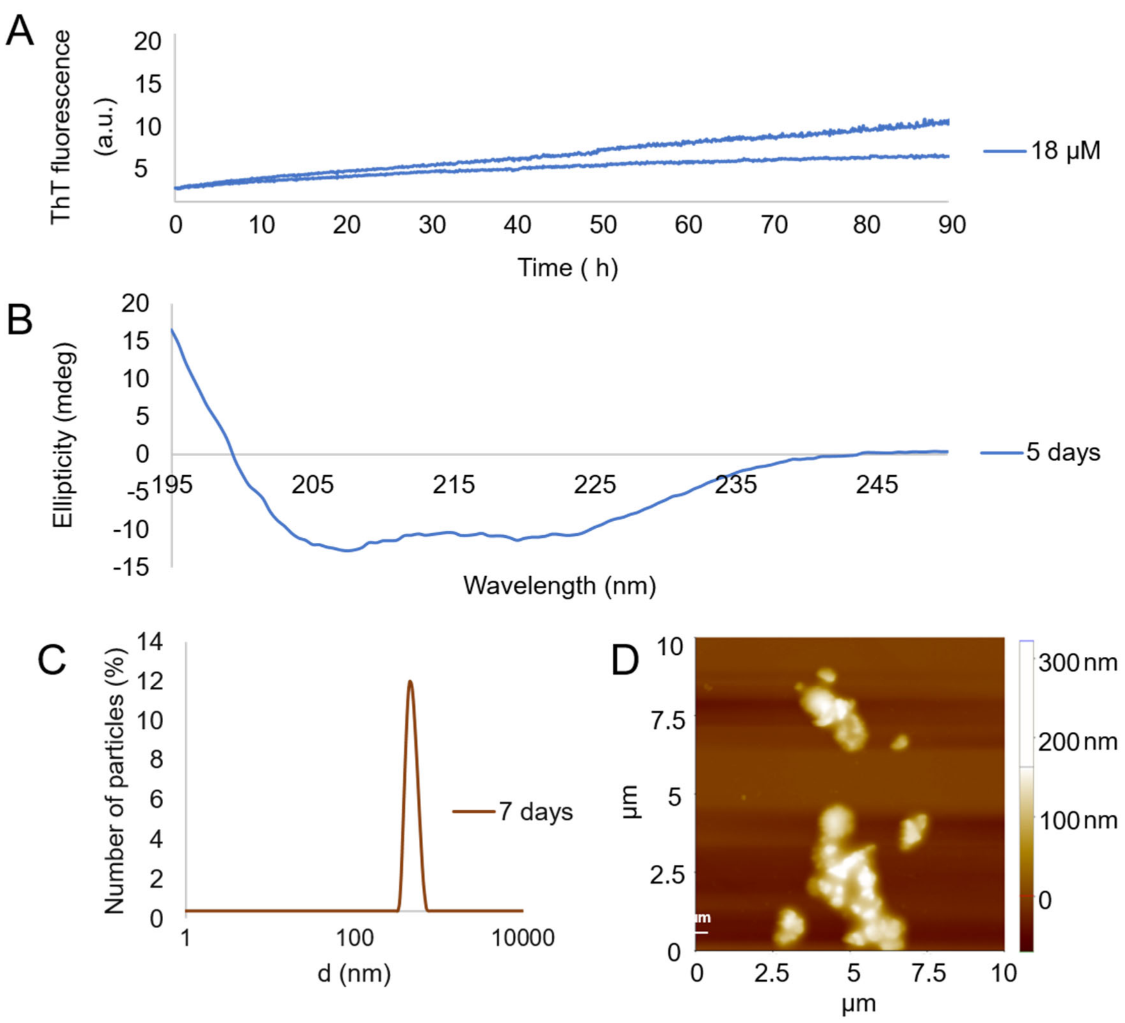

Figure S6. The aggregation of oxidized wt ApoA-I at pH 6.0. A) ThT fluorescence intensity as a function of time measured at 37 °C in duplicate with agitation at 500 rpm for 240 s before each reading of the plate, total cycle time 300 s. B) CD spectrum obtained after 5 days of agitation at 650 rpm in an Eppendorf tube. C) DLS measurement after 6 days of agitation at 650 rpm in an Eppendorf tube, shown as the median of three measurements. D) AFM image after 8 days of agitation at 650 rpm in an Eppendorf tube.

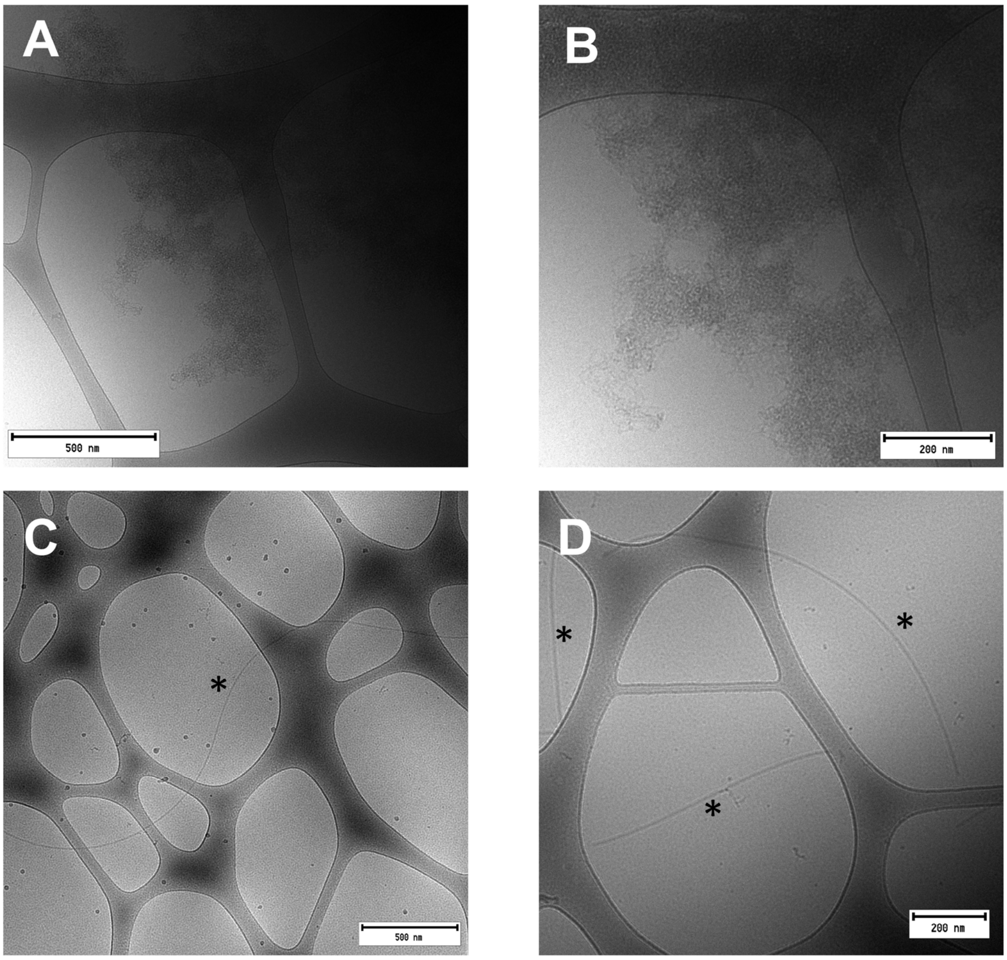

Figure S7: Cryo-TEM of oxidized K107Δ ApoA-I. The sample was aggregated in 20 mM phosphate buffer at pH 6.0, 37 °C. These are additional images to Figure 4, showing the co-existence of larger, more compact aggregates (A-B image), as well as single fibrils (C-D). Please not the difference in magnification of the panels.
